# Tenofovir-induced osteopenia and hyperparathyroidism: A case report and literature review

**DOI:** 10.3389/fendo.2022.1043954

**Published:** 2023-01-11

**Authors:** Jing Zeng, Peng Ye, Dong Wei, Lan Li, Wanxia Ma

**Affiliations:** Department of Endocrinology and Metabolism, Chengdu Second People’s Hospital, Chengdu, Sichuan, China

**Keywords:** tenofovir disoproxil fumarate, osteopenia, hyperparathyroidism, 25-hydroxy vitamin D, chronic viral hepatitis B

## Abstract

Tenofovir disoproxil fumarate is the first-line antiviral therapy for chronic viral hepatitis B, but long-term use is associated with renal failure and hypophosphatemic osteomalacia. Tenofovir disoproxil fumarate-induced osteoporosis and secondary hyperparathyroidism are less commonly reported. Herein, we describe the case of a patient with bone and multijoint pain who was initially misdiagnosed as having normocalcemic primary hyperparathyroidism associated with prolonged exposure to tenofovir disoproxil fumarate. The patient’s 24-h urinary calcium and phosphorus excretion levels and serum calcium levels were at the lower end of the normal range. After reviewing these findings, the diagnosis was amended to osteoporosis and secondary hyperparathyroidism caused by tenofovir disoproxil fumarate. In this report, we describe the differences in clinical and laboratory manifestations of hyperparathyroidism induced by tenofovir disoproxil fumarate and normocalcemic primary hyperparathyroidism. We also discuss relevant pathophysiological mechanisms and propose a feasible treatment strategy.

## Introduction

Chronic viral hepatitis B is widespread in China and requires long-term antiviral treatment. Tenofovir disoproxil fumarate (TDF) is the first-line therapy for chronic viral hepatitis B, but long-term use causes potential kidney and bone damage, resulting in bone aches, muscle weakness, fractures, reduced mobility, and a reduced quality of life (1). There are only a few reports of osteoporosis and secondary hyperparathyroidism resulting from long-term TDF treatment. Herein, we describe a case of osteoporosis and hyperparathyroidism accompanied by prolonged exposure to TDF. We explain the differences between TDF-induced hyperparathyroidism and normocalcemic primary hyperparathyroidism in terms of clinical and laboratory manifestations. We also discuss relevant pathophysiological mechanisms and propose a feasible treatment strategy.

## Case presentation

A 48-year-old postmenopausal woman was admitted to the hospital with shoulder and neck pain that had gradually increased over the last 9 years, pain in her lower back, and multijoint pain (including shoulder joints, elbow joints, hand joints, hip joints, and knee joints) that had emerged over the past year. The pain worsened after physical activity and, for the past year, was accompanied by dizziness, headache, nausea, and numbness of the lips. There was no dry mouth, polydipsia, polyuria, tetany, skin photoallergy, recurrent oral ulcers, or height loss. She had been diagnosed with vitamin D deficiency and osteoporosis 1 year prior at another hospital and had been given four intramuscular injections of ergocalciferol as well as oral calcium carbonate tablets. She had stopped taking the calcium carbonate tablets the previous month. She had been receiving long-term treatment for chronic viral hepatitis B for the last 9 years, which consisted of entecavir at 0.5 mg/day and TDF at 300 mg/day. She had undergone a hysterectomy for cervical cancer 9 years prior to the current presentation and the cancer had not recurred. There was no history of other drug use and no history of fractures. Her family and genetic history were normal.

**Figure 1:**
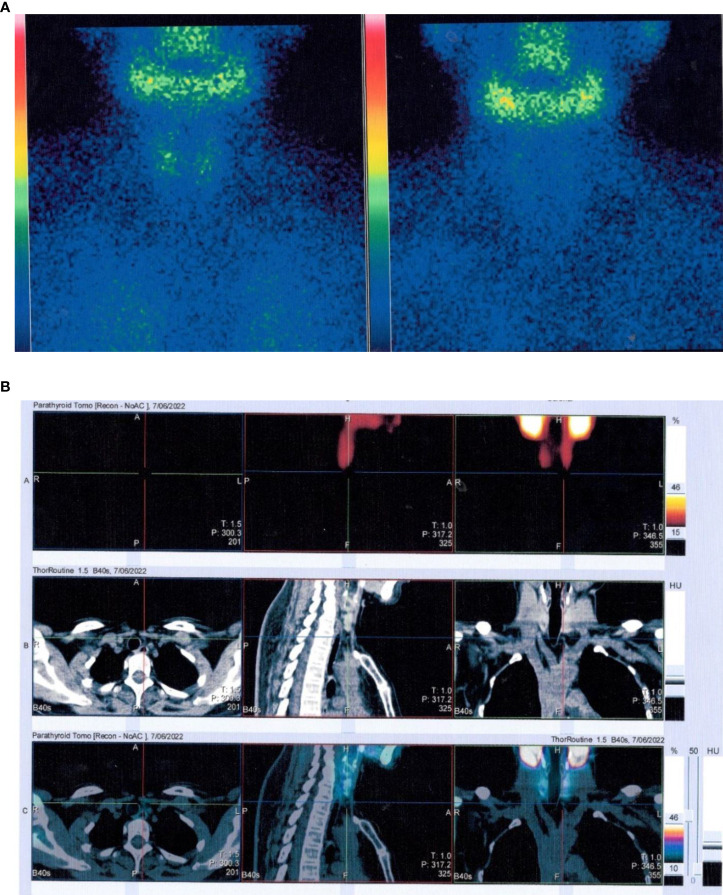
Emission computed tomography of the parathyroid gland. **(A)** No radioactive uptake in the thyroid/parathyroid gland. **(B)** The nodule (8 mm * 3 mm * 5 mm) in the lower pole of the left lobe of the thyroid gland did not uptake radiation.

The patient was 158 cm tall, weighed 47 kg, and had a body mass index of 18.8 kg/m^2^. Her gait, spine curvature, and joints were normal. Tapping the five to seven lumbar vertebrae caused slight pain. There were increased levels of various bone metabolism markers, including alkaline phosphatase, osteocalcin, β-cross-linked C-terminal telopeptide of type I collagen, and procollagen type I N-peptide. Parathyroid hormone (PTH) was also significantly elevated. Serum calcium, serum phosphorus, 25-hydroxyvitamin D, liver tests, renal tests, routine urine tests, serum protein immunofixation electrophoresis, autoimmune antibodies, rheumatoid factors, cancer biomarkers, and thyroid function were all normal. Estradiol was 10.00 pg/ml, progesterone was 0.14 ng/ml, the follicle-stimulating hormone was 85 mIU/ml, the luteinizing hormone was 48.14 mIU/ml, testosterone was 15.81 ng/dl, and prolactin was 12.72 ng/ml (**Table 1**). A kidney ultrasound revealed a renal stone in the left kidney. Thyroid ultrasound indicated a slightly hyperechoic nodule (8 mm × 3 mm × 5 mm) in the lower pole of the left lobe of the thyroid gland, which was considered to be a parathyroid nodule. Dual-energy X-ray absorptiometry was conducted in the lumbar spine (0.629 g/cm^2^, T-score −2.7) and femoral neck (0.804 g/cm^2^, T-score −2.6) and revealed reduced bone mineral density (BMD). Magnetic resonance imaging of the cervical and lumbar vertebrae, as well as an X-ray of both hip joints, indicated no signs of fracture. The preliminary diagnosis was normocalcemic primary hyperparathyroidism. Further tests, including emission computed tomography of the parathyroid gland, revealed no radioactive uptake in the nodule, however (**Figure 1**), and 24-h urinary calcium and phosphorus excretion levels were at the lower end of the normal range, as was the serum calcium level. These findings ruled out primary hyperparathyroidism, vitamin D deficiency, hypophosphatemic osteomalacia, and other causes of osteoporosis. Osteoporosis and secondary hyperparathyroidism caused by TDF were considered. TDF was switched to tenofovir alafenamide at 25 mg/day, combined with cholecalciferol at 800 IU/day and alendronate at 70 mg once a week. The patient’s lower back and joint pain were relieved 1 month later. The PTH level increased in the first month due to the use of alendronate, then decreased slowly over the next 2 months. No hypercalcemia or new renal stones were observed, and the patient was followed up.

**Table 1:** Laboratory examinations before and after treatment.

	Pretreatment	1 month	3 months	Reference
ALT (U/L)	35			7–40
Cr (μmol/L)	59			41–73
Ca (mmol/L)	2.17–2.29	2.28	2.34	2.11–2.52
P (mmol/L)	1.09–1.17	1.23	1.21	0.85–1.51
24hUCa (mmol/L)	3.0			2.5–7.5
24hUP (mmol/L)	14.9			12.9–42.0
25-OHD (ng/ml)	32.14	30.20	35.2	>30
PTH (pg/ml)	87.2–115.0	142	82.5	15–65
ALP (U/L)	99–111		65	35–100
PINP (µg/L)	81.50		69.2	16.27–73.87 (postmenopause)
β-CTX(pg/ml)	850.00		417.67	104–1,008 (postmenopause)
FT3 (pmol/L)	4.33			2.43–6.01
FT4 (pmol/L)	13.13			9.0–19.0
TSH (μIU/ml)	1.560			0.35–4.94
LH (mIU/ml)	48.14			5.16–61.99 (postmenopause)
FSH (mIU/ml)	85			26.72–133.41 (postmenopause)
E2 (pg/ml)	10.00			<28

ALT, alanine aminotransferase; Cr, creatinine; Ca, serum calcium; P, serum phosphorus; 25-OHD, 25-hydroxyvitamin D; PTH, parathyroid hormone; ALP, alkaline phosphatase; PINP, procollagen type I N-peptide; β-CTX, β-cross-linked C-terminal telopeptide of type I collagen; FT3, free triiodothyronine; FT4, free thyroxine; TSH, hormothyrin; LH, luteinizing hormone; FSH, follicle-stimulating hormone; E2, estradiol; 24hUCa, 24-h urinary calcium; 24UP, 24-h urinary phosphorus.

## Discussion

TDF and adefovir dipivoxil, both belonging to the nucleotide reverse transcriptase inhibitor family, are known to potentially cause renal failure, tubule-interstitial diseases, and osteoporosis. The risk is lower with TDF than with adefovir dipivoxil. Postmarketing surveillance reports indicate that the prevalence of severe renal failure caused by TDF is low (< 0.6%), as is that of tubular disease caused by TDF (0.1%) (2). However, the risk of BMD loss is significantly increased with TDF treatment. In a cross-sectional study that included 56,660 participants, there was a 12% increase in the risk of fracture every year associated with prescriptions containing TDF (3). Several studies indicate that TDF is associated with osteoporosis and fracture risk in the lumbar and hip regions. In patients treated with TDF, lumbar BMD decreased by 2.2%–2.57% from baseline and hip BMD decreased by 2.51%–2.8% (4, 5); 42.5% of patients developed osteopenia, and 31.2% of patients developed osteoporosis (5). Notably, increased levels of bone metabolism markers and PTH have been detected in patients shortly after commencing TDF-containing antiretroviral therapy (6), which was associated with BMD loss at 24 weeks (7). This elevated PTH is difficult to distinguish from normocalcemic hyperparathyroidism in the early stages of the disease. To the best of our knowledge, this case report is the first to assess the difference between secondary hyperparathyroidism and osteoporosis induced by TDF and osteoporosis induced by primary hyperparathyroidism in terms of pathophysiological mechanisms and clinical manifestations.

Elevated parathyroid levels can occur in the early stages of TDF treatment (8), and severe 25-hydroxyvitamin D deficiency at baseline increases the risk of secondary hyperparathyroidism (9). Notably, however, significantly elevated PTH levels are also observed in patients with sufficient levels of 25-hydroxyvitamin D (10, 11). The underlying mechanisms are not well understood. TDF’s pharmaceutical toxicity substantially reduces the kidney’s synthesis of active vitamin D. It can also increase the level of the vitamin D binding receptor by 26%, and total 1,25-dihydroxyvitamin D levels, thereby reducing free 1,25-dihydroxyvitamin D (the active form) levels by 42%. This functional vitamin D deficiency can lead to reduced intestinal calcium absorption. Reduced serum calcium increases PTH concentrations, stimulating the conversion of 25-hydroxyvitamin D to 1,25-dihydroxyvitamin D to maintain calcium absorption (12). In addition, TDF can inhibit the activity of the calcium-sensing receptor (CaSR) in a dose-dependent manner (13). Reduced CaSR activity leads to reduced calcium sensitivity in the parathyroid glands and kidneys. As a result, higher serum calcium is required to inhibit excessive PTH release, with increased renal tubular reabsorption of calcium ions and decreased urinary calcium excretion (14). Thus, as a consequence of functional 25-hydroxyvitamin D depletion and suppressed CaSR, TDF-induced hyperparathyroidism is characterized by elevated levels of PTH and bone metabolism markers. However, serum calcium and 24-h urinary calcium excretion levels are at the lower end of the normal range, even in the presence of normal 25-hydroxyvitamin D levels and renal function. These are the main differences in clinical manifestations between normocalcemic primary hyperparathyroidism and secondary hyperparathyroidism induced by TDF.

TDF can cause reduced BMD through direct effects (effects of drugs on osteoclasts and/or osteoblasts) and indirect effects (effects of drugs on proximal renal tubules and/or vitamin D metabolism) (15). It can accumulate in proximal tubular epithelial cells and damage them *via* mitochondrial toxicity (16) and glucose metabolic reprogramming (17). Proximal tubular dysfunction causes calcium and phosphorus resorption disorders and a reduction in the synthesis of active vitamin D in the kidney, leading to the inhibition of bone mineralization. TDF also inhibits the release of adenosine triphosphate and reduces the level of extracellular adenosine, as well as reducing the inhibitory effects of adenosine and adenosine A2A receptors on osteoclasts, increasing osteoclast differentiation, and accelerating bone loss in mouse models and human cell lines (18). Moreover, TDF exposure induces reduced expression of COL1A1 and ATF4 in differentiated primary human osteoblasts, resulting in a significant dose-dependent decrease in mineralization, which suggests that TDF impairs osteoblast mineralization (19).

In the current patient, low body weight was a risk factor for osteoporosis. TDF plasma trough concentrations are significantly higher and exposure time is longer in the context of low body weight (< 50 kg), leading to an increase in adverse events related to TDF, including loss of BMD (20). In a recent cross-sectional study in China, age > 50 years, body mass index < 18.5, and TDF-based treatment were independent risk factors for low BMD (21). In the present patient, another potential risk arose from the combination of TDF and entecavir, which she had been taking for 9 years. The metabolism of TDF can be modified when it is used in combination with other drugs. As an example, when TDF is coadministered with the protease inhibitors ledipasvir or sofosbuvir, plasma TDF exposure increases by 40%–98%, which can aggravate renal damage (22). In recent studies investigating the combination of TDF and entecavir, no significant increases in TDF plasma concentration or the risk of drug-related adverse events were reported (23, 24), but the safety of long-term combination treatment remains unknown. Considering the current patient’s low body weight, we recommend caution with combination therapy and close monitoring of TDF plasma concentrations and renal and bone function.

There is no clear consensus on whether individuals receiving antiretroviral therapy containing TDF should be switched to a more bone-friendly therapy. In patients with a moderate or high risk of fracture, a change to antiviral drugs with non-nucleotide reverse transcriptase inhibitors and protease inhibitors is recommended. Some small-scale studies suggest that BMD in the lumbar spine and femoral neck can improve 48 weeks after TDF treatment is switched to a different treatment regimen (25–27). These studies suggest that the decrease in BMD when TDF therapy is initiated may be largely reversible. In addition, tenofovir alafenamide treatment can be considered, which has lower bone and kidney toxicity with the same antiviral effect (5). Recent studies indicate that when HIV patients receiving TDF treatment are switched to tenofovir alafenamide treatment, their serum levels of 25-hydroxyvitamin D, serum calcium, and PTH normalize, and that the reduced level of serum PTH is dose-dependent (28). Furthermore, the administration of oral high-dose vitamin D3 (50,000–100,000 IU monthly or 4,000 IU daily) also contributes to the reduction of PTH levels (11) and bone loss (29, 30). However, whether Asian individuals can tolerate such high doses of vitamin D is debatable. Experts from the HIV consensus proposal (31) recommend that patients at a high risk of fracture (brittle fracture, osteoporosis, bone loss, or an elevated FRAX score) avoid using nucleotide reverse transcriptase inhibitors and protease inhibitors and instead implement diet and lifestyle management strategies such as calcium supplementation (500–600 mg/day), cholecalciferol (800–1200 IU/day), and anti-osteoporosis therapy. Although bisphosphonates can reportedly improve BMD (32, 33), the use of teriparatide (34) and denosumab (35) has only been reported in a few cases.

## Conclusion

Herein, we have described the case of a patient with TDF-associated osteoporosis and secondary hyperparathyroidism who was treated using vitamin D3 and bisphosphonates after a change in antiretroviral medication. After the new treatment regimen, the patient exhibited a slow reduction in PTH levels and relief from pain. Long-term follow-up is still required, however, to assess subsequent changes in BMD and the parathyroid nodule. TDF is the first-line treatment for chronic viral hepatitis B. The long-term use of TDF has been linked to increased risks of osteoporosis, fracture, and secondary hyperparathyroidism, but these potential hazards have received little clinical attention. The cause of this elevated PTH is difficult to distinguish from normocalcemic hyperparathyroidism in the early stages. Close monitoring of TDF plasma concentrations and renal and bone function is necessary. Early detection, diagnosis, and treatment of osteoporosis and secondary hyperparathyroidism induced by these drugs should be emphasized.

## Data availability statement

The original contributions presented in the study are included in the article/supplementary material. Further inquiries can be directed to the corresponding author.

## Ethics statement

The studies involving human participants were reviewed and approved by the Research Ethics Committee of the Chengdu Second People’s Hospital. The patients/participants provided their written informed consent to participate in this study. Written informed consent was obtained from the individual(s) for the publication of any potentially identifiable images or data included in this article.

## Author contributions

JZ and PY were responsible for the collection of the clinical data and treated the patient in the inpatient department. LL and WM were responsible for the collection of the clinical data and the follow-up of the patient in the clinic. DW designed and revised the manuscript. All authors contributed to the article and approved the submitted version.
